# Uric acid and metabolic syndrome: Findings from national health and nutrition examination survey

**DOI:** 10.3389/fmed.2022.1039230

**Published:** 2022-12-14

**Authors:** Rodney G. Bowden, Kathleen A. Richardson, Luke T. Richardson

**Affiliations:** ^1^Department of Public Health, Baylor University, Waco, TX, United States; ^2^Department of Health, Human Performance and Recreation, Baylor University, Waco, TX, United States; ^3^ProtaGene US, Inc., Burlington, MA, United States

**Keywords:** NHANES, metabolic syndrome, uric acid, hyperuricemia, cardiovascular disease

## Abstract

**Introduction:**

Hyperuricemia commonly associated with Gout has been proposed as an independent risk factor for Metabolic Syndrome (MetS).

**Objective:**

The purpose of the study was to determine if there is a relationship between hyperuricemia and MetS.

**Methods:**

An analysis of cross-sectional data was conducted using the 2013–2018 National Health and Nutrition Examination Survey (NHANES) datasets. Sample weights were assigned by NHANES researchers to each participant allowing researchers to generalize results to all non-institutionalized United States (US) civilians. The analysis included 6,432 individuals, which were representative of 94,729,059 US citizens.

**Results:**

Pearson’s correlations, chi-square tests, and logistic regression equations were calculated to determine the association between hyperuricemia and MetS. In an unadjusted regression analysis, individuals with hyperuricemia (above 7.0 mg/dL in males and 6.0 mg/dL in females) were 3.19 times more likely to have MetS compared to those with normal uric acid (UA) levels. When controlling for various confounding variables those with hyperuricemia were 1.89 and 1.34 times more likely to have MetS than those with normal UA levels in two additional logistic regression models.

**Conclusion:**

In this large cross-sectional study, hyperuricemia was found to be associated with MetS. Additional analyses that controlled for various risk factors previously identified as predictive of MetS still demonstrated hyperuricemia independently associated with MetS. The results of this study suggest a need to understand the metabolic pathways of UA more clearly to further explain the contribution to MetS. Additional research should include prospective clinical trials assessing the effects of UA and the control of UA on MetS and concomitant medical outcomes.

## Introduction

Cardiovascular disease (CVD) has historically been associated with developed, wealthy, and industrialized nations primarily affecting higher socioeconomic individuals. Most recently, this leading cause of death has become endemic in developing countries as well (1). Metabolic syndrome (MetS) is a clustering of risk factors that are primarily associated with chronic diseases such as CVD, type II diabetes, hypertension, stroke and contributes to over 15 million deaths a year globally (2). Additional comorbid conditions associated with MetS include dyslipidemia, increases in blood glucose, central adiposity, and insulin resistance (3, 4). MetS continues to increase globally with significant increases in the last two decades and is considered one of the most significant risk factors for CVD (3, 5). The International Diabetes Federation estimates that 25% of the global population has MetS with rates varying based on age, gender and ethnicity (6). Identifying a novel biomarker for MetS to complement existing biomarkers could help identify more at-risk people and help gain a deeper understanding of the complex pathogenesis of MetS (3).

Uric acid (UA) has been postulated as a novel biomarker for MetS and is associated with the end-stage of purine degradation. (6) Normal levels of UA provide a protective effect against free-radical oxidative damage (3). UA is formed by the liver, is excreted by kidneys with some production by the intestines (7). Hyperuricemia (above 7.0 mg/dL in men and 6.0 mg/dL in women) has been reported in previous studies (3–7) to be associated with many of the same biomarkers of MetS and diseases such as diabetes, CVD, and kidney disease. Many studies have suggested hyperuricemia is an established risk factor for MetS, (8–10) while others have not reported a relationship when comparing those with and without Diabetes Mellitus (DM) (11). Study authors have also reported increased prevalence of MetS in patients with hyperuricemia when compared to healthy populations with an increase between 30 and 41% in patients with gout (12–15). It should be noted that gout is a common inflammatory disease associated with hyperuricemia. Therefore, the purpose of the study was to compare a large cohort of participants from the National Health and Nutrition Examination Survey (NHANES) with and without MetS and their corresponding UA levels to understand if hyperuricemia is associated with MetS.

## Materials and methods

The present study utilizes data from the 2013 to 2018 NHANES datasets. The data were acquired through the Center for Disease Control and Prevention (CDC) website (16). The study was determined exempt from IRB review by the sponsoring university due to the nature of the secondary data analysis (IRB ID# 1505514-1).

### Study sample

The NHANES sample represents civilian, non-institutionalized United States (US) citizens. The sample design includes multi-year, stratified, clustered four-stage samples that are published in 2-year cycles on the CDC website. Underrepresented groups, such as ethnic minorities, the impoverished, children, and older adults, are oversampled to obtain more precise subgroup estimates. The present study utilizes complex survey sample weighting procedures outlined by the NHANES analytic guidelines (17–19). In the NHANES sampling procedure, sample weights are calculated and assigned to everyone in the sample. Weighting considers the known probability of selection, non-responders, and variations between the sample and the US population. Sample weighting allows researchers to produce results that would have been obtained if the entire US population were surveyed. Using this method, the statistics produced for a single individual in the NHANES sample are extrapolated to be representative of many persons in the greater US population.

In the present study we analyzed three 2-year cycles of NHANES which spanned 2013–2018. We began with 29,400 subjects in those three cohorts. Subjects on dialysis in the past 12 months (*n* = 59), were reported to be or tested positive for being pregnant (*n* = 190), individuals younger than 18 or older than 79 (*n* = 12,585), those not having all the data for MetS (*n* = 10,111) and did not have UA data (*n* = 23) were excluded from the study for a final sample size of 6,432. Individuals between the ages of 18 and 79 were included for analysis as the upper age limit was set because subjects’ ages in the NHANES dataset are top coded at 80 years to prevent identification. When the survey sample weighting techniques were utilized, the subsample was representative of 94,729,059 US citizens.

### Demographic and variable information

The procedure documents for the NHANES questionnaires, exams, and laboratory tests are outlined on the CDC website (20). Trained reviewers administered questionnaires to subjects using a computer-assisted interview system. Socioeconomic status (SES) was considered “low” for individuals who fell at or below 100% of the poverty line. Subjects were classified as smokers if they reported tobacco use within the 5 days prior to the study or if they reported smoking more than 100 cigarettes in their entire life. Subjects were considered physically active if they engaged in 75 or more minutes of vigorous-intensity recreational physical activity, 150 min or more of moderate-intensity physical activity, or an equivalent combination of both (21, 22). International Classification of Diseases, tenth revision (ICD-10) codes were used to classify prescription drug information gathered *via* questionnaires for the study. “Glucose Medication” included any prescription medication used to treat hyperglycemia (codes R73, E11, E11.2, E11.2P, E11.4, and E11.P), “Cholesterol Medication” included any medication used to treat dyslipidemia (codes E78.0, E78.0P, and E78.1), “Hypertension Medication” included any medication used to treat hypertension (codes I10 and I10.P), and “Hyperuricemia Medication” included any medication used to treat hyperuricemia (codes E79.0, M10.9, M10.9P, and M1A).

Hyperuricemia was defined as a UA level >7.0 mg/dL in men or >6.0 mg/dL in women. The harmonized definition of by Alberti et al. (23) was used to categorize metabolic risk factors (24). Risk factors include: elevated waist circumference (≥102 cm in males, ≥88 cm in females, ≥90 cm in Asian males, or ≥80 cm in Asian females), elevated triglycerides (≥150 mg/dL) or prescription drug treatment for elevated triglycerides, reduced High Density Lipoprotein Cholesterol (HDL-c) (<40 mg/dL in males or <50 mg/dL in females) or prescription treatment for reduced HDL-c, elevated blood pressure (systolic ≥130 mmHg and/or diastolic ≥85 mmHg) or prescription treatment for hypertension, and elevated fasting glucose (≥100 mg/dL) or prescription treatment for hyperglycemia. MetS and “metabolically unhealthy” were defined by three or more metabolic risk factors. Renal function was calculated by the CKD-EPI equation (23).

### Statistical analyses

All statistical analyses were conducted in SAS version 9.4 (SAS Institute Inc., Cary, NC, USA). Normality of variables was assessed using measures of skewness and kurtosis and by visual inspection of histograms, P-P, and Q-Q plots. Unweighted, continuous demographic variables were reported as mean ($\bar{\text{x}}$) and standard deviation (SD) whereas weighted variables were reported as mean ($\bar{\text{x}}$) and standard error (SE). Unweighted, categorical demographic variables were reported as frequency (n) and percentage (%) and weighted variables were reported as percentage (%) and SE. Survey sample weights were assigned to demographic variables and statistical tests using survey procedures in SAS (PROC SURVEY). Individuals meeting the inclusion criteria for the study were included in weighted analyses using a domain statement, which ensured accuracy in the number of elements in the sample and SE values. Differences in categorical variables were assessed using χ2 tests whereas simple regression was used to determine statistical differences between continuous variables. Pearson’s correlation coefficient was calculated to determine the correlation between metabolic risk factors and UA levels. Sample weighting was used in logistic regression models to determine if there is a relationship between hyperuricemia and MetS Probability values were considered significant at the α < 0.05 level. All analyses utilize survey sample weighting unless otherwise noted.

## Results

**FIGURE 1:**
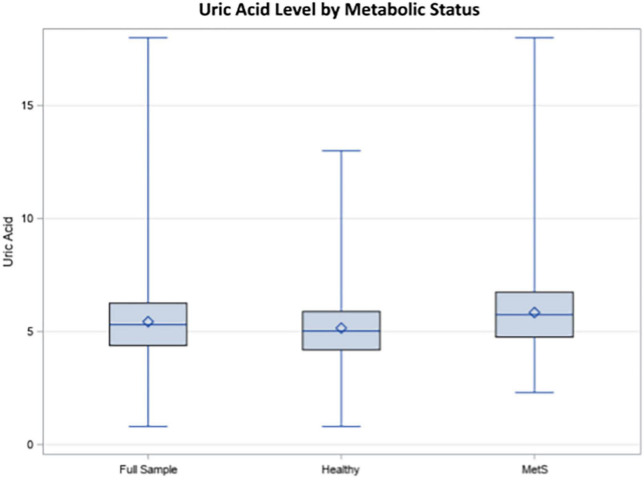
Uric acid (UA) levels in the total sample, healthy individuals, and individuals with metabolic syndrome (MetS).

Demographic information for the sample is reported in Table 1. Hyperuricemia was present in 17.66% of individuals in the study and the average UA level was 5.44 mg/dL in the overall weighted sample. Individuals with hyperuricemia had an average UA level of 7.64 mg/dL, whereas those with normal UA levels averaged 5.00 mg/dL (*p* < 0.0001). As demonstrated in Figure 1, metabolically healthy individuals had an average UA level of 5.15 mg/dL and hyperuricemia was present in 10.69% of the metabolically healthy individuals, whereas 27.83% of individuals with MetS had hyperuricemia (*p* < 0.0001) and the average UA level was 5.84 in those with MetS. Prescription medication for gout and/or hyperuricemia was reported in 1.20% of the overall sample. There were a significantly greater number of people with CKD in the hyperuricemia group. In those with normal UA, 5.54% had CKD and MetS. In those with hyperuricemia, 17.78% had CKD and MetS.

**TABLE 1:** Demographic information for the total sample, those with hyperuricemia, and those with normal uric acid (UA) levels.

	Unweighted total (*n* = 6,432)	Weighted total (*n* = 94,729,059)	Hyperuricemia (17.66%)	Normal UA (82.34%)	*P*-value
	$\bar{\text{x}}$ (SD) or *M* (IQR)	$\bar{\text{x}}$ (SE)	$\bar{\text{x}}$ (SE)	$\bar{\text{x}}$ (SE)	
Age (years)	46.91 (17.01)	46.18 (0.38)	48.97 (0.72)	45.58 (0.41)	** < 0.0001**
Uric acid (mg/dL)	5.46 (1.44)	5.44 (0.02)	7.46 (0.04)	5.00 (0.02)	** < 0.0001**
Fasting glucose* (mg/dL)	101(94,111)	107.90 (0.48)	111.66 (1.13)	107.10 (0.50)	**0.0003**
Triglycerides* (mg/dL)	92(62,138)	115.00 (1.69)	149.03 (4.62)	107.68 (1.59)	** < 0.0001**
HDL* (mg/dL)	51(42,62)	54.42 (0.37)	49.43 (0.76)	55.50 (0.36)	** < 0.0001**
Systolic blood pressure (mmHg)	123.30 (17.93)	121.57 (0.30)	126.14 (0.75)	120.59 (0.30)	** < 0.0001**
Diastolic blood pressure (mmHg)	70.16 (12.23)	70.44 (0.30)	72.12 (0.57)	66.78 (0.34)	** < 0.0001**
Waist circumference (cm)	99.37 (17.16)	99.93 (0.43)	110.50 (0.918)	87.46 (0.35)	** < 0.0001**
BMI (kg/m^2^)	29.34 (7.21)	29.34 (0.18)	33.55 (0.37)	28.43 (0.17)	** < 0.0001**
HOMA-IR*	2.47(1.48,4.31)	3.83 (0.10)	5.28 (0.32)	3.51 (0.09)	** < 0.0001**
hs-CRP* (mg/L)	1.90(0.80,4.46)	3.75 (0.18)	5.28 (0.45)	3.42 (0.18)	**0.0003**
BUN* (mg/dL)	13(10,16)	13.88 (0.12)	15.73 (0.22)	13.49 (0.12)	** < 0.0001**
SCr* (mg/dL)	0.83(0.69,0.98)	0.86 (0.00)	0.97 (0.01)	0.84 (0.00)	** < 0.0001**
eGFR (ml/min/1.73 m^2^)	97.76 (22.17)	96.61 (0.50)	87.93 (0.93)	98.48 (0.51)	** < 0.0001**
ALT* (IU/L)	20(15,28)	24.76 (0.28)	29.93 (0.86)	23.65 (0.24)	** < 0.0001**

	***n* (%)**	**% (SE)**	**% (SE)**	**% (SE)**	***p*-value**

Male sex	3140 (48.82)	49.71 (0.70)	48.13 (0.79)	57.08 (2.13)	**0.0005**
**Race/ethnicity**					
Mexican American	1007 (15.66)	9.43 (1.13)	7.04 (1.19)	9.94 (1.19)	**0.0063**
Other Hispanic	720 (11.19)	6.52 (0.80)	5.58 (0.88)	6.72 (0.86)	
NH white	2292 (35.63)	63.48 (1.99)	64.84 (2.70)	63.18 (1.93)	
NH black	1334 (20.74)	11.24 (1.11)	13.32 (1.59)	10.80 (1.05)	
NH Asian	824 (12.81)	5.50 (0.53)	6.05 (0.74)	5.38 (0.55)	
Other/multi-racial	255 (3.96)	3.84 (0.41)	3.17 (0.76)	3.99 (0.43)	
Low SES	1307 (22.45)	15.28 (1.06)	15.19 (1.48)	15.29 (1.10)	0.9358
MetS	2792 (43.41)	40.36 (1.07)	63.60 (2.19)	35.38 (1.04)	** < 0.0001**
CKD	929 (14.44)	11.91 (0.51)	22.55 (1.47)	9.63 (0.50)	** < 0.0001**
Physically active	2274 (69.88)	69.22 (1.09)	65.78 (2.40)	69.88 (1.32)	0.1702
Smoker	2897 (45.04)	46.34 (1.27)	53.12 (1.95)	44.88 (1.41)	**0.0005**
Glucose medication	766 (11.91)	9.03 (0.54)	15.07 (1.30)	7.73 (0.57)	** < 0.0001**
Cholesterol medication	1159 (18.02)	17.07 (0.68)	22.79 (1.51)	15.85 (0.68)	** < 0.0001**
Hypertension medication	1612 (25.06)	21.80 (0.87)	37.02 (1.83)	18.53 (0.90)	** < 0.0001**
Hyperuricemia medication	78 (1.21)	1.20 (0.19)	2.35 (0.55)	0.96 (0.22)	**0.0117**

*P*-value is representative of a significant difference between those with hyperuricemia and those with normal UA values. Simple regression was used for continuous variables and chi-square tests were used for categorical variables to determine the statistical differences between the hyperuricemia group and the normal UA group. GFR was estimated by the CKD-EPI equation. HDL, high-density lipoprotein cholesterol; HOMA-IR, homeostatic model assessment for insulin resistance; hs-CRP, high-sensitivity C-reactive protein; BUN, blood urea nitrogen; SCr, serum creatinine; eGFR, estimated glomerular filtration rate; ALT, alanine aminotransferase; SES, socioeconomic status; MetS. *Variable is not normally distributed in unweighted sample; median and interquartile range (Q1, Q3) are reported rather than mean and SD.

On average, individuals with hyperuricemia had 4.56 mg/dL higher levels of blood glucose (*p* = 0.0003), 41.35 mg/dL higher levels of triglycerides (*p* < 0.0001), 5.55/2.04 mmHg higher blood pressure (*p* < 0.001), 12.84 cm greater waist circumference (*p* < 0.0001), and 1.86 mg/dL higher hs-CRP (*p* = 0.0003) as compared to those with normal UA levels. HDL was 6.07 mg/dL lower (*p* < 0.0001) and eGFR was 10.55 ml/min/1.73 m^2^ lower in the hyperuricemia group (*p* > 0.0001) compared to the normal UA group. Those with hyperuricemia were more likely to be male and non-Hispanic (NH) White, NH Black, or NH Asian. have MetS, smoking, and prescription medications for hyperglycemia, dyslipidemia, hypertension, and hyperuricemia were more frequently reported in those with hyperuricemia. There was no statistical difference in SES and self-reported physical activity level between the hyperuricemia and normal UA groups (*p* = 0.936 and 0.170, respectively).

The five metabolic risk factors were found to have small to moderate significant correlations to UA levels in the overall group, demonstrated in Table 2. When exclusively analyzing metabolically healthy individuals, all metabolic risk factors were significantly correlated with UA level with the exception of fasting blood glucose (*r* = 0.0.24, *p* = 0.146). In individuals with MetS, fasting glucose, triglycerides, HDL, and waist circumference levels were found to have small to moderate correlations, whereas blood pressure was not significantly correlated to UA.

**TABLE 2:** Pearson’s correlations with uric acid (UA) levels using an unweighted sample.

	Total group (*n* = 6,432)	Healthy	MetS
Fasting glucose	*r* = 0.038, *p* = 0.002	*r* = 0.024, *p* = 0.146	*r* = −0.094, *p* < 0.0001
Triglycerides	*r* = 0.176, *p* < 0.0001	*r* = 0.225, *p* < 0.0001	*r* = 0.083, *p* < 0.0001
HDL	*r* = −0.278, *p* < 0.0001	*r* = −0.238, *p* < 0.0001	*r* = −0.194, *p* < 0.0001
Systolic BP	*r* = 0.164, *p* < 0.0001	*r* = 0.157, *p* < 0.0001	*r* = 0.011, *p* = 0.555
Diastolic BP	*r* = 0.105, *p* < 0.0001	*r* = 0.115, *p* < 0.0001	*r* = 0.021, *p* = 0.273
Waist circumference	*r* = 0.339, *p* < 0.0001	*r* = 0.283, *p* < 0.0001	*r* = 0.241, *p* < 0.0001

The individual correlation values (Pearson’s r) are reported for the correlations between UA and the metabolic risk factor variables. Values are reported for the total, healthy, and MetS groups. BP, blood pressure.

The logistic regression models in Table 3 demonstrate that individuals with hyperuricemia are more likely to have MetS. In an individual with hyperuricemia, the unadjusted odds of having MetS are 3.19 (*p* < 0.0001), demonstrated in Model 1. After adjustment for potentially confounding variables, the relationship was attenuated (OR = 1.88, *p* < 0.0001 in Model 2 and OR = 1.38, *p* = 0.297 in Model 3). The strength of the logistic regression models was improved with each addition of potential confounders.

**TABLE 3:** Logistic Models demonstrating the odds of having metabolic syndrome (MetS).

	Model 1				Model 2				Model 3			
		95% CI for odds ratio				95% CI for odds ratio				95% CI for odds ratio		
Coefficient	B (SE)	Lower	OR	Upper	B (SE)	Lower	OR	Upper	B (SE)	Lower	OR	Upper
Intercept	−0.60*(0.05)	–	–	–	−7.62*(0.50)	–	–	–	−8.29*(0.69)	–	–	–
**Hyperuricemia (yes)**	1.16*(0.08)	2.69	3.19	3.79	0.63*(0.14)	1.42	1.88	2.49	0.32 (0.26)	0.81	1.38	2.37
Age					0.06*(0.00)	1.06	1.07	1.07	0.06*(0.01)	1.05	1.06	1.07
Sex–Male					0.15 (0.12)	0.91	1.16	1.49	0.06 (0.20)	0.71	1.06	1.58
Race–Mexican American					−0.03(0.14)	0.73	0.97	1.30	−0.13(0.23)	0.55	0.88	1.39
Race–Hispanic					−0.25(0.17)	0.55	0.78	1.11	−0.28(0.30)	0.41	0.75	1.38
Race–NH black					−0.50*(0.14)	0.45	0.61	0.81	−0.41(0.22)	0.42	0.67	1.05
Race–NH Asian					0.78*(0.13)	1.69	2.19	2.84	0.94*(0.20)	1.71	2.56	3.82
Race–multi-racial					0.30 (0.23)	0.84	1.36	2.19	0.56 (0.30)	0.95	1.76	3.24
hs-CRP					0.00 (0.01)	0.98	1.00	1.03	−0.01(0.02)	0.95	0.99	1.03
BMI					0.10*(0.02)	1.07	1.10	1.14	0.10*(0.02)	1.06	1.11	1.16
HOMA-IR					0.37*(0.04)	1.32	1.45	1.58	0.41*(0.07)	1.31	1.51	1.74
Smoking									0.31 (0.16)	0.98	1.37	1.90
Physical activity									−0.29(0.21)	0.49	0.75	1.14
BUN									0.01 (0.02)	0.97	1.01	1.06
SCr									0.39 (0.39)	0.67	1.48	3.27
ALT									0.01 (0.01)	1.00	1.01	1.02
UA drug (yes)									1.41 (0.84)	0.74	4.09	22.70
Cox and snell *R*^2^	0.046				0.377				0.375			
Nagelkerke *R*^2^	0.062				0.507				0.516			
Model χχ^2^	361.26, *p* < 0001				170.16, *p* < .0001				52.56, *p* < 0001			

Model 1: Unadjusted logistic regression model with MetS (yes/no) as outcome variable. Model 2: Logistic regression model adjusted for known risk factors associated with MetS (age, sex, race, inflammation, body mass index, and HOMA-IR score). Model 3: Logistic regression model adjusted for known risk factors associated with MetS (age, sex, race, inflammation, body mass index, and HOMA-IR score, smoking status, physical activity, blood urea nitrogen, serum creatinine, alanine transaminase, and hyperuricemia drug). Model comparisons are made with *R*^2^ and model χ^2^ values. *p < 0.01.

## Discussion

The purpose of the study was to determine if there was a relationship between hyperuricemia and MetS. Our study findings suggest that hyperuricemia had significant statistical and clinical associations with MetS in participants in the NHANES study. These findings partially support previous studies that reported similar findings (15, 25, 26) but does not support previous studies that did not discover a relationship (11). Those participants with hyperuricemia (>7.0 mg/dL in males and >6.0 in females) in our study were more than three times as likely to have concomitant MetS when compared to those with normal UA levels. When controlling for variables associated with hyperuricemia and MetS, participants with hyperuricemia were still 1.38–1.88 times more likely to have MetS than without hyperuricemia, suggesting that there is a relationship between hyperuricemia and MetS. Understanding the role of UA in MetS can help to identify additional risk for MetS and can help elucidate possible metabolic pathways associated with CVD and other chronic diseases.

Various studies (1–10) have reported a relationship between UA and MetS, supporting the findings of our study. Mahajan et al. (7) reports that UA raises blood pressure levels through the stimulation of intracellular oxidative stress. Additionally, hyperuricemia may increase insulin resistance, vasodilation, increase blood flow, interfere with nitric oxide function, and therefore affect glucose absorption. Previous studies (8, 25, 26) postulate that UA has an antioxidant effect at normal levels but becomes proinflammatory in people with hyperuricemia and increased oxidative stress. Our study findings support this previous research as hs-CRP was significantly and clinically lower in the normal UA group. A previous study (7) suggested that hyperuricemia may cause inflammatory responses in adipose tissues causing increased inflammation and insulin resistance and promote fat storage. Participants in our study in the hyperuricemia group have significantly larger waist circumferences, supporting the previous study findings. These higher levels of inflammation can also have concomitant changes in lipids, blood pressure and kidney function, increasing the risk for other chronic conditions, which is supported by our study.

It should be noted that some previous studies did not support our findings of hyperuricemia being associated with MetS. Those studies that did not support our results primarily involved a small number of participants (11) or utilized Mendelian randomization that failed to show a causal relationship (12, 16) or made comparisons between those with a singular risk factor for MetS (11, 16). Pfister et al. (11) conducted a case-control study of 7,504 patients with diabetes and 8560 controls without diabetes. Their findings did not support an association with UA and DM, but did not make comparisons on other known risk factors for MetS. Adnan et al. (16)reported on findings from a study of 102 primarily older participants focusing a component of MetS rather than all risk factors for MetS as well, in an observational study that was conducted over 3 months. Additionally, Copur et al. (12) reported that previous studies that did not find associations between UA and MetS mostly used Mendelian randomization methods, which was not used by our study. Sample characteristics and findings of each study can be found in Table 4.

**TABLE 4:** Summary of similar studies regarding UA and MetS.

References	Sample size	Study population	Methodology	Findings*
El Aziz et al. (3)	52	26 obese children (6–9 years of age) with MetS 26 obese children (6–9 years of age) without MetS	Prospective case-control	Positive correlations in: BMI, WC, SBP, DBP, FBG, triglycerides, TC, LDL Negative correlation: HDL
Galindo-Yllu et al. (4)	292	202 women, 90 men hospital health personnel in Peru (46.2 years of age + 10.6)	Cross-sectional analytical	Positive association in MetS and UA, primarily in women. UA an independent risk factor for IR and Triglycerides
Baygi et al. (5)	234	Adult male seafarers working on seafarers (36 years of age + 10.3)	Cross-sectional	UA associated with MetS, high TG, high TC
Khan et al. (6)	360	180 cases with CAD 180 controls without CAD (51.37 years of age ± 6.5 years)	Case-control	Positive association between UA and CAD
Mahajan et al. (7)	200	100 cases with MetS (53.53 years of age + 12.14 100) controls without MetS (50.12 years of age + 11.64)	Case-control	Hyperuricemia in 64% of patients with MetS and 20% of patients without MetS
Al Shanableh et al. (8)	871	Adults between 18 and 40 years of age in Qatar Biobank database	Cross-sectional	Asymptomatic high UA associated with prediabetes, dyslipidemia, and subclinical inflammation
Pfister et al. (11)	10,080	7,562 cases with DM 8,560 controls without DM EPIC-Norfolk cohort study database Age range 40–79 years	Mendelian Randomization	No association between UA and Type II Diabetes
Ali et al. (14)	420	257 men and 163 women (30.52 years of age + 12.4)	Cross-sectional	UA significantly associated with the prevalence of MetS
Shi-Jiao et al. (31)	3,808	3,808 female Jinchuan non-ferrous Metals Corporation workers (40–60 years of age) pre and post-menopausal	Cross-sectional	Correlation between UA levels and MetS association was stronger in premenopausal women
Adnan et al. (16)	102	Gender not reported (51.5 years of age + 12.4)	Observational cross-sectional	IR and MetS associated study. UA and MetS not associated
Majeed and Hashim et al. (26)	100	50 cases with MetS, 50 controls without Mets, 56 males and 40 females. Age of participants not provided	Descriptive case-control	UA associated with MetS

*BMI, body mass index; WC, waist circumference; SBP, systolic blood pressure; DBP, diastolic blood pressure; FBG, fasting blood glucose; TC, total cholesterol; LDL, low density lipoprotein; HDL, high density lipoprotein; MetS, metabolic syndrome; UA, uric acid; IR, insulin resistance; TG, triglycerides; CAD, coronary artery disease.

A thorough review of the literature by Caliceti et al. (27) suggests that increasing UA levels associated with MetS may be attributed to increased levels of fructose in the Western diet, which has been adopted in many countries. The increase in fructose is normally associated with increases in added sugars in both food and drink. The corresponding hyperuricemia is caused primarily by the enzyme xanthine oxidase. (28) Though a causal link has yet to be established, further research is needed to further elucidate these findings (29, 30).

Patients from our study with hyperuricemia had significantly elevated levels of most risk factors associated with MetS as noted in Table 1, except for HDL-C which had lower levels in the hyperuricemia group. The only risk factors that were not significantly different included physical activity levels and SES. These findings suggest that UA may be both a risk factor for MetS yet additionally related to other MetS risk factors, CVD, and other chronic conditions such as chronic kidney disease. The correlations in Table 2, while weak to moderate in strength, represent a statistically significant influence of UA on each of the metabolic risk factors. These findings suggest UA could be providing additional risk for the development of MetS.

Findings from the logistic regression in our study suggest that hyperuricemia has a significant relationship with MetS, and when controlling for known risk factors of MetS, those with hyperuricemia were still more likely to have concomitant MetS. In Model 3, the most statistically adjusted model, the results were attenuated, indicating that the relationship between hyperuricemia and MetS was weakened after controlling for additional risk factors. Yet, this still suggests that those with hyperuricemia are more likely to have MetS based on the OR findings.

Inferences from this study are limited due to the cross-sectional nature of the data collection which limited the ability to make causal inferences. Single time point measures occur with NHANES data collection, and the study is therefore not longitudinal. Additionally, some data collected by NHANES surveys are self-reported. However, the sample is a large and diverse sample collected by CDC that is both healthy and unhealthy participants making analyses and findings of importance. Statistical analysis allowed for the sample to be representative of the greater population of the US, yielding sound, generalizable results.

Findings from the logistic regression in our study reported in Table 3 suggests that hyperuricemia has an association with MetS, and when controlling for known risk factors of MetS, those with hyperuricemia were still more likely to have MetS. Further research is needed to better understand the role UA plays in the development of MetS and to elucidate metabolic pathways associated with UA and MetS.

## Data availability statement

Publicly available datasets were analyzed in this study. This data can be found here: National Health and Nutrition Examination Survey https://www.cdc.gov/nchs/nhanes/index.htm.

## Ethics statement

This study was determined exempt from IRB review by the sponsoring university due to the nature of the secondary data analysis (IRB ID# 1505514-1).

## Author contributions

RB and KR created the research study idea, participated in the study design, methodology, wrote the manuscript, and proofed the final manuscript. KR analyzed the data and reported the findings from the results of the study. LR assisted in the analysis of the data, wrote the manuscript, and proofed the final version of the manuscript. All authors contributed to the article and approved the submitted version.
